# Commensal Bacteria and MAMPs Are Necessary for Stress-Induced Increases in IL-1β and IL-18 but Not IL-6, IL-10 or MCP-1

**DOI:** 10.1371/journal.pone.0050636

**Published:** 2012-12-07

**Authors:** Thomas Maslanik, Kate Tannura, Lucas Mahaffey, Alice Brianne Loughridge, Lida Benninson, Luke Ursell, Benjamin N. Greenwood, Rob Knight, Monika Fleshner

**Affiliations:** 1 Department of Integrative Physiology and the Center for Neuroscience, University of Colorado at Boulder, Boulder, Colorado, United States of America; 2 Department of Chemistry and Biochemistry, and the Howard Hughes Medical Institute, University of Colorado at Boulder, Boulder, Colorado, United States of America; University of California, Riverside, United States of America

## Abstract

Regular interactions between commensal bacteria and the enteric mucosal immune environment are necessary for normal immunity. Alterations of the commensal bacterial communities or mucosal barrier can disrupt immune function. Chronic stress interferes with bacterial community structure (specifically, α-diversity) and the integrity of the intestinal barrier. These interferences can contribute to chronic stress-induced increases in systemic IL-6 and TNF-α. Chronic stress, however, produces many physiological changes that could indirectly influence immune activity. In addition to IL-6 and TNF-α, exposure to acute stressors upregulates a plethora of inflammatory proteins, each having unique synthesis and release mechanisms. We therefore tested the hypothesis that acute stress-induced inflammatory protein responses are dependent on the commensal bacteria, and more specifically, lipopolysaccharide (LPS) shed from Gram-negative intestinal commensal bacteria. We present evidence that both reducing commensal bacteria using antibiotics and neutralizing LPS using endotoxin inhibitor (EI) attenuates increases in some (inflammasome dependent, IL-1 and IL-18), but not all (inflammasome independent, IL-6, IL-10, and MCP-1) inflammatory proteins in the blood of male F344 rats exposed to an acute tail shock stressor. Acute stress did not impact α- or β- diversity measured using 16S rRNA diversity analyses, but selectively reduced the relative abundance of *Prevotella.* These findings indicate that commensal bacteria contribute to acute stress-induced inflammatory protein responses, and support the presence of LPS-mediated signaling in stress-evoked cytokine and chemokine production. The selectivity of the commensal bacteria in stress-evoked IL-1β and IL-18 responses may implicate the inflammasome in this response.

## Introduction

The enteric mucosal immune system is a unique immunological site that must maintain a balance between responding to harmful pathogens and avoiding inappropriate immune responses to food or symbiotic bacteria. During a brief developmental period, ecological secession culminates in a relatively stable community of commensal bacteria [1]. Regular interactions between the mucosal immune system and these bacteria are critical for proper regulation of mucosal as well as systemic immune function [2]–[4]. Moreover, disruptions to the mucosal environment such as changes in barrier function or microbial composition can lead to severely dysregulated immunity [5], [6].

Several diverse factors may impact the mucosal barrier or the composition of the commensal bacteria including antibiotic use [7], [8], changes to diet or hygiene [7], [8], and activation of the stress response [9]–[14]. Dense sympathetic innervation of the intestine [15], and stress-inducible, localized mast cell degranulation [5], could facilitate stress-evoked changes to both the composition of the commensal bacteria [9], [11], [16], [17] and the integrity of the intestinal barrier [13], [18], [19]. Importantly, stress-induced changes to the intestinal barrier or the composition of the commensal bacteria appear to drive some aspects of stress-evoked mucosal and even systemic immune activity. Stress-induced disruptions to the mucosal barrier, for example, are linked to increased serum cytokine levels including tumor necrosis factor α (TNFα) [20]. Similarly, reducing the commensal bacteria via antibiotic administration attenuates chronic or repeated stress-induced enhancements in splenic macrophage activity [18] and circulating levels of the cytokine interleukin-6 (IL-6) [1].

Exposure to stressors, however, evokes a broad cytokine and chemokine response beyond the few cytokines that manipulations to the mucosal environment have been shown to modulate. Stress, for example, increases circulating concentrations of several inflammatory proteins including not only TNFα and IL-6, but also interleukin-1β (IL-1β) [21]–[23], interleukin-18 (IL-18) [21], interleukin-10 (IL-10) [24], and monocyte chemotactic protein-1 (MCP-1) [25]–[27]. Importantly, these and other cytokines operate in networks with other inflammatory proteins to achieve immunological effects [24]. Moreover, activation, synthesis, release, and mechanisms of various stress-responsive cytokines and chemokines are different and could vary in their modulation by the intestinal bacteria. Multiple stress-responsive cytokines must therefore be considered when investigating the role of intestinal bacteria in stress-induced alterations in immune activation.

Furthermore, previous studies implicating changes to the enteric mucosal immune system in stress-evoked immune activity focus on chronic or repeated stressors such as social defeat or repeated restraint [1]. These stressors not only activate the stress response, but can produce long-term changes to metabolic processes [28], feeding [29], and grooming behavior [30], which could themselves influence immune function or the role of intestinal bacteria in stress-evoked immune activation. Stress-evoked cytokine and chemokine secretion occurs in response to acute stressors. Thus the acute stress response itself might affect the production of these cytokines independent of other stress-evoked long-term adaptations. Understanding the role of commensal bacteria in the acute stress-induced production of a broad range of inflammatory proteins could provide important new information about how stress affects specific immunological pathways.

We therefore tested the hypothesis that acute stress-induced immune modulation depends on commensal bacteria. We reduced commensal bacteria using antibiotics, exposed rats to an acute tail shock stressor, and measured cytokine and chemokine production. Alterations in gut microbiota composition can influence immune function. A second goal was to test if exposure to an acute stressor would produce changes in microbiota diversity measured using 16S rRNA diversity analyses. Finally, the mechanism by which the commensal bacteria communicate with the immune system during stressor exposure, including acute stressor exposure, remains unknown. LPS, a microbe-associated molecular pattern (MAMP), is found in the cell membrane of some commensal bacteria and can increase in the circulation [19] following intestinal barrier disruption as occurs with chronic stress. Thus a third goal was to determine whether LPS is an important signaling molecule for communication between commensal bacteria and the immune system. We administered endotoxin inhibitor (EI) to block LPS, and measured circulating cytokines and chemokines after acute stress. Our results make several novel contributions to the literature in that they reveal an important role for intestinal bacteria in acute stress-induced immune activation, and support the presence of LPS-mediated signaling from the commensal bacteria in stress-induced cytokine and chemokine production. Furthermore, the results may reveal details of the signaling pathway underlying stress-evoked cytokine and chemokine production and could support the future development of therapeutics designed to manipulate stress-induced immune activity.

## Methods

### Subjects and Housing

Adult male Fischer 344 rats (240–260 g) were divided equally into four groups crossing stress and antibiotic (N = 64) or stress and EI administration (N = 32). The Fischer Rat is a highly stress responsive inbred rat, and was chosen for these experiments as the stress response is robust and consistent across animals allowing us to use fewer animals per group. To characterize the impact of stress on the commensal bacteria, rats (N = 25) were divided into 3 groups to examine the effect of stress both immediately and 24 hours following stressor termination. All rats were maintained on a 12∶12-h light-dark cycle (lights on from 0700 to 1900) in a specific pathogen free environment. Animals were allowed two weeks to acclimate to the colony room prior to any experimental manipulation. Rats were handled briefly each day for 1 week before the start of the study. All animals were housed in Plexiglas Nalgene cages and allowed *ad libitum* access to food (Harlan Laboratories, Denver, CO) and water. Colony room temperature was maintained at 23°C. The care and treatment of the animals were in accordance with protocols approved by the University of Colorado Institutional Animal Care and Use Committee.

### Stress

On the day of the experiment, animals either remained in their home cages (*Control*) or were exposed to 100, 1.5 mA, 5-second, intermittent, (average trial interval = 60 seconds+/−25 seconds) inescapable tail shocks (*Stress*) as previously described [24], [35]–[37]. During the stress procedure, rats were placed in a Plexiglas restraining tube (23.4 cm long, 7 cm diameter). Electrodes were then placed across the tail that protruded from the back of the shock tube. The shocks were administered by an automated shock system (Precision Calculated Animal Shocker; *Colbourne* Instruments). This tail shock procedure is a well-established model of acute stress that has been thoroughly characterized in terms of both the stress response and the immune response. To examine the role of the commensal flora in an immune response to an acute stressor we selected this model of stress for its robust impact on immune function [24], [31]–[34]. *Stress* occurred between 0730 and 1130 to avoid differences in cytokine and chemokine production due to circadian rhythms. Immediately after termination of stress, all animals were sacrificed via rapid decapitation unless otherwise noted.

### Quantification of the stress response

Because the duration, intensity, and chronicity of a stressor determines the immunological consequences of a stressor exposure [35], [38], [39], and because the absence of the commensal bacteria in germ-free rodents modulates HPA responses after stress [40], we measured two important markers of activation of the stress response to provide a characterization of tail shock. Corticosterone and spleen weights were measured to demonstrate activation of the stress response. Corticosterone is a measure of hypothalamic-pituitary-adrenal axis (HPA) output, and reductions in spleen weight are directly proportional to sympathetic nervous system activity. Corticosterone was measured in 96-well microtiter plates using commercially available ELISAs in accordance with manufacturer's instructions (Enzo Life Sciences). Optical densities were measured using a SpectraMax Plus 354 plate reader (Molecular Devices) and concentrations were analyzed using a four-parameter curve fitting software (SoftMax 5.4.1). Spleens were harvested aseptically and weighed immediately.

### Antibiotic Administration

For 4 days prior to *Stress* rats received either drinking water plus 4.0 mg/ml streptomycin and 2.0 mg/ml penicillin g (antibiotic) or drinking water alone (water) *ad libitum* as previously described [41], [42]. Antibiotics were administered in the drinking water to avoid the potential stress-response associated with other delivery methods such as oral gavage [43], [44]. The current antibiotic regimen was selected for its broad-spectrum antibacterial effects and because it is consumed by the rats without the addition of any flavoring or sweetener. Each morning, antibiotic solution was replaced because penicillin G has a short half-life at room temperature. Water bottles were weighed daily to estimate water intake of all rats and ensure equivalent doses between animals. Body weights were recorded and fecal matter was examined to monitor sickness or diarrhea in rats receiving antibiotics.

### Endotoxin Inhibitor Administration

On the day of *Stress,* EI was prepared by dissolving 1.0 mg/ml of EI into sterile PBS, which was stored on ice in the dark until use. Fifteen minutes prior to *Stress,* rats received an intraperitoneal injection (i.p.) of either 1.0 mg/kg EI (Bachem) or PBS alone. This dose was adapted from previous investigations demonstrating that this concentration of EI was sufficient to reduce LPS activity [45], [46]. The time between the injection of EI and the start of *Stress* was necessary to achieve maximal efficacy of the drug based upon the short half-life of EI.

### Sample Collection

Immediately following sacrifice, whole blood was collected in EDTA coated vacutainers using a polypropylene funnel and centrifuged at 3000×g for 15 minutes at 4°C to obtain plasma samples. Fecal samples were collected from each animal immediately prior to the beginning of *stress* in sterile, media free, dual culture swabs (Becton Dickinson). Additional samples were taken immediately following termination of *stress* and from animals 24 hours following the termination of *stress* in the same manner. Following collection, all samples were frozen at −80°C.

### Quantification of Bacteria

In order to confirm the efficacy of our antibiotic regimen, fresh fecal samples were collected from a subset of rats immediately prior to the beginning of *stress*. These samples were homogenized in 2.0 ml PBS and plated at several dilutions on nutrient agar. Plated samples were allowed to incubate at 37.0°C for 48 hours. Following incubation, colony forming units (CFU) of bacteria were counted, and dilution-corrected averages were recorded. Although many anaerobic bacteria will not grow on nutrient agar, this media was selected because it grows both Gram-positive and Gram-negative bacteria. Because the selected antibiotic regimen is broad spectrum, and targets both aerobic and anaerobic bacteria, reduced CFU counted on nutrient agar confirms effective reduction of commensal bacteria by the antibiotic regimen.

### Endotoxin Measurement

In a separate experiment, whole blood was collected from *Stress* and *Control* rats in endotoxin free tubes. After 1 hr at room temperature, these samples were centrifuged at 3000×g for 15 min at 4.0°C to separate serum. LPS was quantified in serum using a Limulus amebocyte lysate (LAL) assay per manufacturer's instructions (Lonza).

### 16S rRNA microbial community analysis

Fecal samples and cecal contents were collected and prepared for sequencing using previously established protocols [47], [48]. Briefly, the MoBio 96 htp PCR clean up kit was used to triplicate, combine, and clean each sample. The samples underwent the PCR reaction with both forward and reverse primers (F515/R806) to target the V4 variable region of the 16S rRNA. The reverse primer contained an error-correcting 12-base Golay code allowing correct demultiplexing of ∼1,500 samples even when sequencing introduces errors in the barcode region. After gel purification and ethanol precipitation to remove PCR artifacts, a composite sample containing equimolar ratios of the amplicons were sequenced with the Illumina HiSeq 2000 (average sequences per sample 34,664±13,577 standard deviation (SD)). The open-source software package QIIME 1.3.0 [49] was used to process the sequences and conduct statistical analysis. Sequences were clustered into operational taxonomic units (OTUs) based on 97% sequence similarity using Uclust [50]. Taxonomy was assigned to OTUs using the Ribosomal Database Project classifier [51] against the GreenGenes 16S rRNA database [52].

Alpha diversity of samples was assessed with the Phlyogenetic Diversity metric, and results confirmed with Chao1 and observed species metrics (data not shown). Each sample was randomly subsampled 10 times, without replacement, at sequence depths from 2,000 to 30,000 sequences per sample at steps of 2,000 sequences per sample. The error bars on graphs depicting alpha diversity measures indicate the range of alpha diversity values at each sampling depth. ANOVA was used to determine whether any bacterial taxa significantly changed in abundance as a result of stress, with a p-value <0.05 following false discovery rate (FDR) correction.

### Cytokine measurement

Circulating concentrations of cytokines (IL-1β, IL-6, IL-10, MCP-1, IL-18, IL-15) were measured from plasma in 96-well microtiter plates using commercially available ELISAs in accordance with the manufacturer's instructions. IL-1β, IL-6, and MCP-1 were measured in ELISAs from R&D Systems. IL-10 and IL-18 were measured in ELISAs from Invitrogen. Optical densities were measured using a SpectraMax Plus 354 plate reader (Molecular Devices) and concentrations were analyzed using a four-parameter curve fitting software (SoftMax 5.4.1).

### Statistical Analyses

A two-tailed independent t-test was used to determine whether antibiotic affected numbers of colony forming units of commensal gut bacteria. Two-way repeated measures analyses of variance (ANOVA) were used to test for differences body weight between all groups of rats. Two-way ANOVAs were run to analyze the effect of stress or antibiotic on individual cytokines and chemokines. Data points were treated as outliers if they failed Grubb's test for outliers [53] and were also recorded as affected by experimental procedures by the experimenter. Data are presented as means ± the standard error of the mean. P<0.05 was considered statistically significant.

## Results

### Single exposure to tail shock activated the stress response

**Figure 1:**
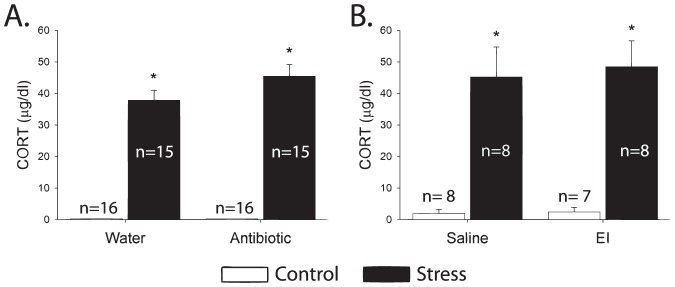
Exposure to an acute stressor significantly increases circulating corticosterone. The increase in circulating corticosterone is typical of acute activation of the hypothalamic-pituitary-adrenal axis such as that which occurs as part of activation of the stress response. Neither antibiotics (**A**) nor endotoxin inhibitor (**B**) impacted the corticosterone response to tail shock. (*p<0.05).

**Figure 2:**
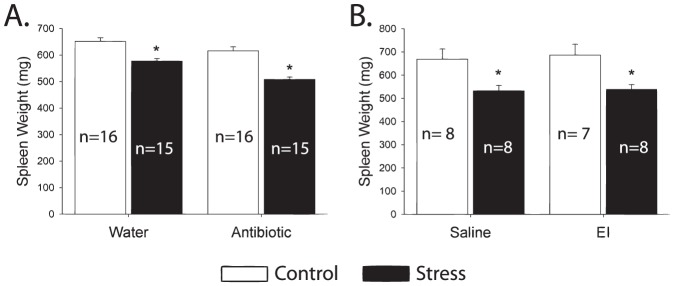
Exposure to an acute stressor causes significant splenic atrophy. This increase is not impacted by either antibiotics (**A**) or endotoxin inhibitor (**B**). Splenic atrophy is typical of acute activation of the sympathetic nervous system such as that which occurs as part of activation of the stress response. (*p<0.05).

Consistent with prior work using this stressor [54]–[56], exposure to acute tail shock stress resulted in increased plasma corticosterone over control levels (p<0.001) (**Figure 1**). Reduced spleen weight, indicating sympathetic nervous system activity [57], [58], was observed following stress independent of antibiotic or EI treatment (p<0.001) (**Figure 2**). These values represent large changes from baseline and are indicative of a severe acute stressor.

### Antibiotic administration effectively reduced commensal bacterial load

**Figure 3:**
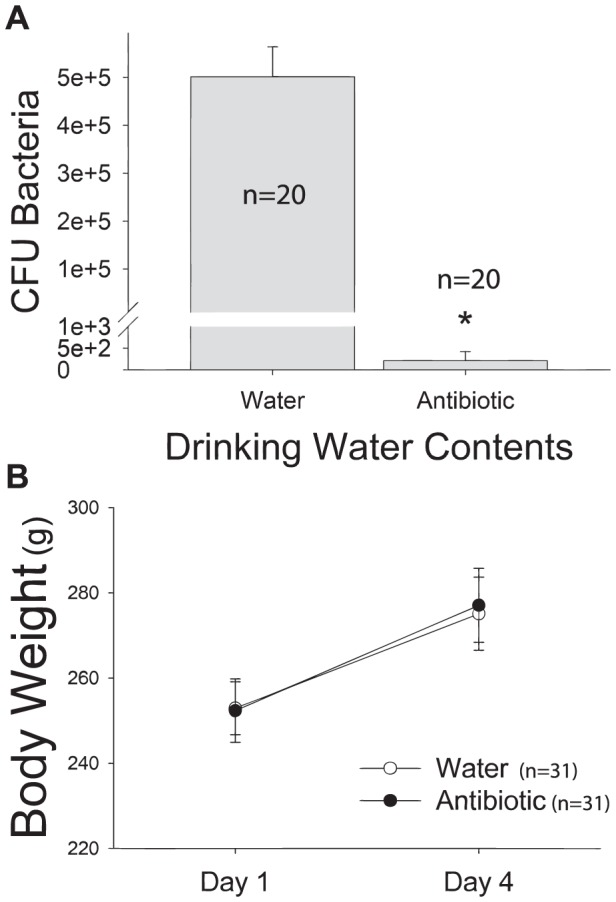
The efficacy of antibiotic administration is shown by successful reduction of colony forming units of bacteria in the absence of gross physiological changes such as a reduction in body weight gain. **Figure 3A** shows colony forming units of bacteria cultured on nutrient agar were significantly reduced in rats receiving antibiotic treatment. Although many species of commensal bacteria cannot be cultured on nutrient agar, the reduction produced by antibiotics is indicative of successful depletion of commensal bacteria by antibiotics, as neither the agar or the antibiotic regimen are specific for any particular group of bacteria. (*p<0.05). **Figure 3B** **:** depicts body weight changes across antibiotic treatment regimen. Body weights increased in all groups across time and were unaffected by antibiotic treatment. (*p<0.05).

The number of colony forming units measured in the fecal samples of rats receiving antibiotics was significantly lower than control rats (p<0.001) (**Figure 3A**). In fact, in all but one rat, the number of CFU observed in rats receiving antibiotics was below the detectable limit. This decrease in CFU count suggests that antibiotic administration significantly reduced the commensal bacteria, as expected. No decrease in body weight was observed, suggesting that antibiotics did not create other gross physiological changes that could confound the interpretation of the results (**Figure 3B**).

### Stress increased circulating LPS

**Figure 4:**
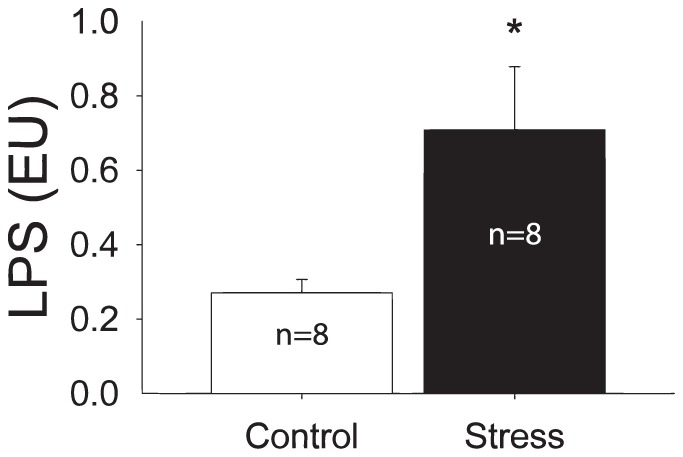
Stress evokes a significant increase in circulating concentrations of LPS. The systemic or circulating increased concentration of LPS is indicative of leakage of the commensal bacteria and their byproducts. (*p<0.05).

Levels of LPS in the circulation are quite low at baseline reflecting adequate barrier function of the mucosal surfaces that contain the commensal flora. Exposure to acute stress increases the concentration of LPS measured in plasma (p<0.01) (**Figure 4**). Levels of LPS measured in the circulation changed from 0.2708±0.0361 EU to 0.7084±0.1700 EU possibly reflecting changes to the mucosal environment in response to stress.

### Acute-stress alters the relative abundance of *Prevotella* but does not impact overall diversity

**Figure 5:**
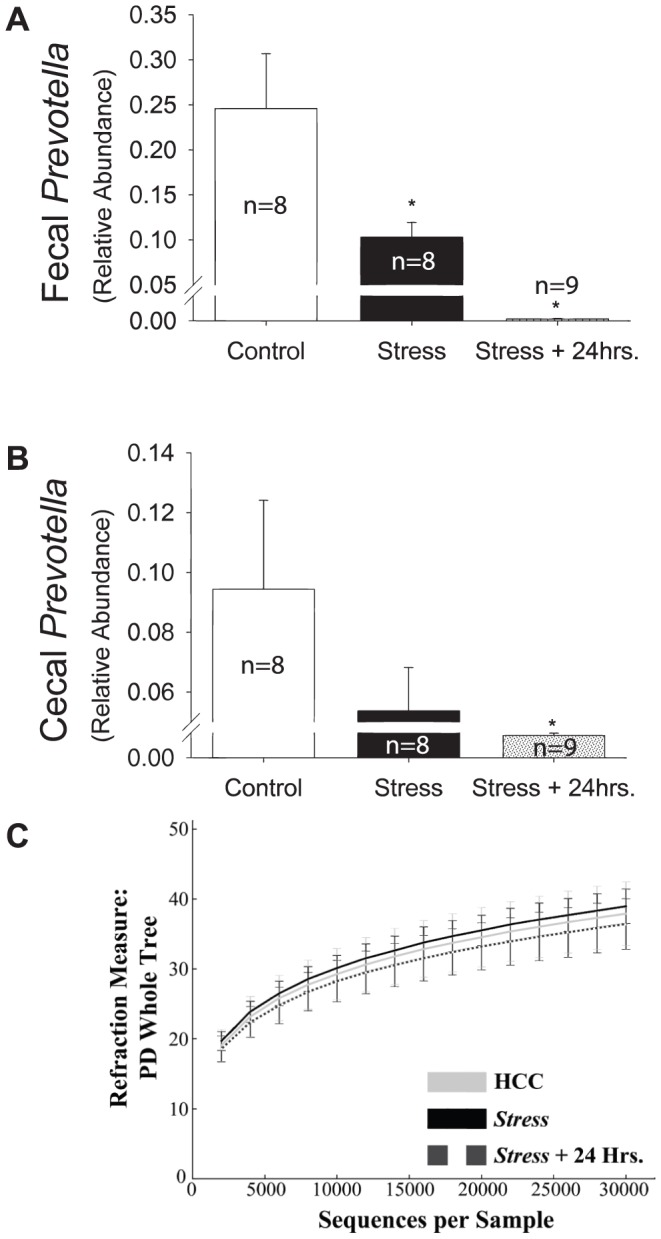
The impact of stressor exposure on the commensal flora. Figure 5A depicts the relative abundance of *Prevotella* decreased in fecal samples immediately following termination of the stressor. These changes persisted for at least 24 hours after the rats were returned to their home cages. (*p<0.05). Figure 5B shows the relative abundance of *Prevotella* decreased in cecal content samples. Although there was an immediate trend following the termination of the stressor, the difference in the relative abundance of *Prevotella* was only significant 24 hours later (*p<0.05). Figure 5C reveals no effect of stress on α-diversity in fecal samples. Cecal samples similarly showed no changes in overall diversity attributable to stress (data not shown).

16S rRNA analysis can reveal the relative abundance of all genera in the microbiome (including genus-level clusters of DNA sequences that have not yet been formally described). Stress caused a decrease in the relative abundance of a single genus, *Prevotella.* The decrease was detectable immediately following stress (p<0.05), and persisted for 24 hours after stressor termination (p<0.01), in fecal samples taken from the colon (**Figure 5a**). The relative abundance of *Prevotella* also decreased in cecal samples (p<0.01), although this decrease was only statistically significant 24 hrs after stressor termination (**Figure 5b**). There was no change α-diversity (the mean species diversity on a local scale, such as within a fecal sample) created by acute tail shock stress (**Figure 5c**). Similarly, stress did not impact β-diversity (differentiation in mean species diversity between collection sites).

### Antibiotic administration attenuated the production of some cytokines

**Figure 6:**
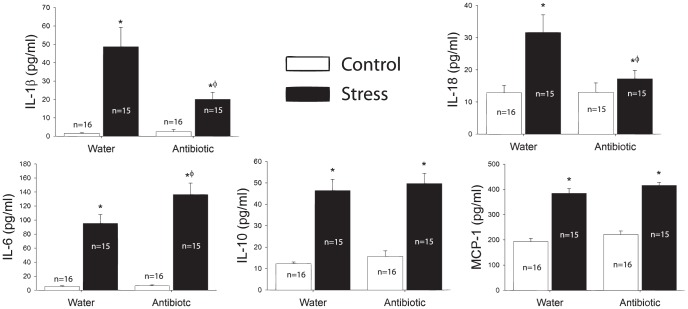
Stress evokes a significant increase in circulating IL-1β, IL-6, IL-10, IL-18, and MCP-1. Administration of antibiotics significantly attenuated the impact of stress on IL-1β and IL-18. Antibiotics, however, did not attenuate IL-10 or MCP-1, and actually increased levels of circulating IL-6. (*p<0.05 vs. control, water; Ф p<0.05 vs. stress, water).

*Stress* increased circulating levels of IL-1β (p<0.001), IL-6 (p<0.001), IL-10 (p<0.001), IL-18 (p<0.001), and MCP-1 (p<0.001) (**Figure 6**). Administration of antibiotics attenuated the stress-induced production of IL-1β (p<0.01) and IL-18 (p<0.05) (**Figure 6**). However, administration of antibiotics failed to attenuate the stress-induced production of IL-6, IL-10, and MCP-1 (**Figure 6**). Interestingly, although antibiotics reduced the impact of stress on some cytokines, they increased circulating levels of IL-6 following stress (p<0.05).

### EI administration attenuated the production of the same cytokines as antibiotics

**Figure 7:**
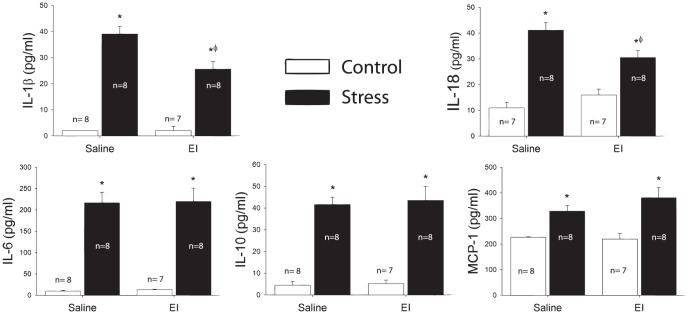
Stress evokes a significant increase in circulating IL-1β, IL-6, IL-10, IL-18, and MCP-1. Administration of endotoxin inhibitor significantly attenuated the impact of stress on IL-1β and IL-18. Endotoxin inhibitor, however, did not attenuate IL-6, IL-10, or MCP-1. (*p<0.05 vs. control, water; Ф p<0.05 vs. stress, water).

*Stress* again increased circulating levels of IL-1β (p<0.001), IL-6 (p<0.001), IL-10 (p<0.001, IL-18 (p<0.001), and MCP-1 (p<0.001) (**Figure 7**). As with antibiotic administration, administration of EI attenuated the stress induced production of IL-1β (p<0.01) and IL-18 (p<0.05) (**Figure 7**). Administration of EI also failed to attenuate the stress-induced production of IL-6, IL-10, and MCP-1 (**Figure 7**).

## Discussion

The results support the hypothesis that commensal bacteria and LPS release contribute to stress-evoked increase in cytokines and chemokines in the blood. Adult male rats exposed to acute tail shock displayed robust elevations in plasma concentrations of several cytokines and chemokines including IL-1β, IL-6, IL-10, IL-18, and MCP-1. The administration of oral antibiotics or endotoxin inhibitor reduced the stress-induced elevation of IL-1β and IL-18, but interestingly, not IL-6, IL-10, or MCP-1. Thus, another signal beyond the commensal bacteria or released LPS is sufficient for synthesis and release of cytokines and chemokines such as IL-6, IL-10, and MCP-1 following stressor exposure.

Chronic stressors can create shifts in commensal bacterial diversity [9], and this change can impact peripheral immunity, as well as render the intestine vulnerable to pathogenic bacteria infection. Our results suggest that although intact commensal bacteria are necessary for stress-evoked increases in IL-1β and IL-18, these increases are not correlated with decreases in overall bacterial community diversity. Exposure to acute tail shock did not impact overall diversity (either α-diversity or β-diversity) in either fecal or cecal samples. Tail shock did, however, reduce the relative abundance of *Prevotella. Prevotella* is a highly prevalent genus of Gram-negative bacteria in the normal commensal bacterial community and has been reported to drive the overall composition of the flora [59]. Changes in the relative abundance of this genus may have broad physiological and immunological consequences. Reduced abundance of *Prevotella* can impact immunological inflammatory disease states such as inflammatory bowel disease [60], eczema [61], and rheumatoid arthritis patients [62]. Thus the stress-induced reduction in *Prevotella* may reduce anti-inflammatory status of the mucosal immune system and potentially contribute to the pro-inflammatory state produced by stress. Alternatively, the stress-induced reduction in *Prevotella* may result from death of these bacteria and which would result in the release of pro-inflammatory MAMPs, such as LPS as observed following stressor exposure.

Another goal of the current studies was to determine the specific role of LPS as a signaling molecule important for communication between commensal bacteria and the immune system. The current data support a signaling pathway involving LPS, released from the commensal bacteria in stress-induced cytokine and chemokine responses. Because stress increased circulating concentrations of LPS, leakage of LPS from the commensal bacteria may be important in stress-induced cytokine and chemokine production. Inhibiting LPS by administering EI produced the same effects as antibiotic administration, reducing stress-induced increases in IL-1β and IL-18 but not attenuating IL-6, IL-10, or MCP-1. Although the magnitude of the EI induced attenuation of stress induced IL-1β and IL-18 production was smaller than that observed with antibiotics, EI only inhibits the signaling action of LPS, whereas antibiotic treatment should also reduce other MAMPs such as peptidoglycans [63]. As in the antibiotic study, another signal was sufficient for stress-induced synthesis and release of IL-6, IL-10, and MCP-1.

The present study goes beyond prior work in several important respects. First, it examined a greater number of cytokines and chemokines than previous investigations, providing a broader view of the immune response. Second, it demonstrated that the commensal bacteria have a role in stress-induced inflammatory protein production following a single exposure to an acute stressor. Third, it described no effect of acute stressor exposure on intestinal diversity and a selective reduction in *Prevotella.* And finally, for the first time, it directly examined the mechanism by which the commensal bacteria (via LPS release) influence stress-induced cytokine and chemokine production. Our results thus reveal a novel role for the gut commensal bacteria in selective modulation of inflammatory proteins. This selectivity of the flora to impact IL-1β and IL-18 may help to fully reveal the mechanisms by which stress and the commensal flora impact immune function.

Recent studies highlight several features unique to the synthesis and release of IL-1β and IL-18 that are not necessary for the production of other cytokines and chemokines such as IL-6, IL-10, or MCP-1 [64]. The unique features in the synthesis pathways of these proteins result in different signaling requirements for these two families of inflammatory proteins [65] and could, thus, explain the selectivity of the antibiotic or EI induced attenuation in the stress-induced cytokine and chemokine response. Of particular importance, while IL-6, IL-10, MCP-1, and the majority of other cytokines and chemokines are synthesized in their releasable form, IL-1β and IL-18 are synthesized as inactive precursors [64], [66]. Processing, therefore, is required in the complete synthesis and release pathway for IL-1β and IL-18. This post-translational processing is predominately mediated by caspase-1, an enzyme activated upon the assembly of a multimeric signaling complex called the inflammasome [67]–[69]. Recent data suggests that the inflammasome is involved in stress-evoked cytokine and chemokine production [70]. Given that antibiotics and EI selectively affect IL-1β and IL-18, these data suggest an interaction between the stress response, the commensal bacteria, and the inflammasome in stress-induced cytokine and chemokine production.

Investigations examining the inflammasome have highlighted the necessity of two signals for inflammasome assembly or activation. The first signal leads to synthesis of components of the inflammasome, as well as pro-IL-1β, and pro-IL-18 [71], [72]. Inflammasome independent cytokines are also completely synthesized in response to a single signal [73]. The second signal leads to final inflammasome assembly and caspase-1 activation [67], [74]. *In vitro,* the requirement for two signals has been demonstrated using a MAMP and a danger associated molecular pattern (DAMP) such as ATP, Hsp72, Uric Acid, or even elevated concentrations of glucose [65], [71], [75]. Administration of a MAMP [65], [76], [77] or DAMP [78] alone is not capable of activating the inflammasome, however, co-administration of both ligands is sufficient for inflammasome and cytokine production.

Although the exact nature of the requirement for the combination MAMPs and DAMPs is unknown, neutralizing only a single MAMP was sufficient to selectively suppress the inflammasome dependent cytokines *in vivo*. Because MAMPs from the commensal bacteria were only necessary for stress-induced synthesis of inflammasome dependent cytokines, MAMPs likely provide the second signal necessary for inflammasome activation. Although speculative, it seems probable that *in vivo* after exposure to an acute stressor, DAMPs likely act as the first signal in stress-evoked cytokine and chemokine production. DAMPs such as Hsp72 and uric acid are known to increase in response to many stressors [79]–[81] including tail shock [55], [56], [70], [82]–[84]. Furthermore, DAMPs, as well as stress-evoked IL-6, IL-10, or MCP-1 responses are not impacted by either antibiotic or EI treatment. Thus, DAMPs may underlie the stress-induced release of the inflammasome independent cytokines and chemokines and hence, act as the first signal in the inflammasomal pathway of stress-evoked cytokine and chemokine production. Other secretions of the stress response such as catecholamines may also act as the first signal in stress-evoked cytokine and chemokine production [85], [86].

**Figure 8:**
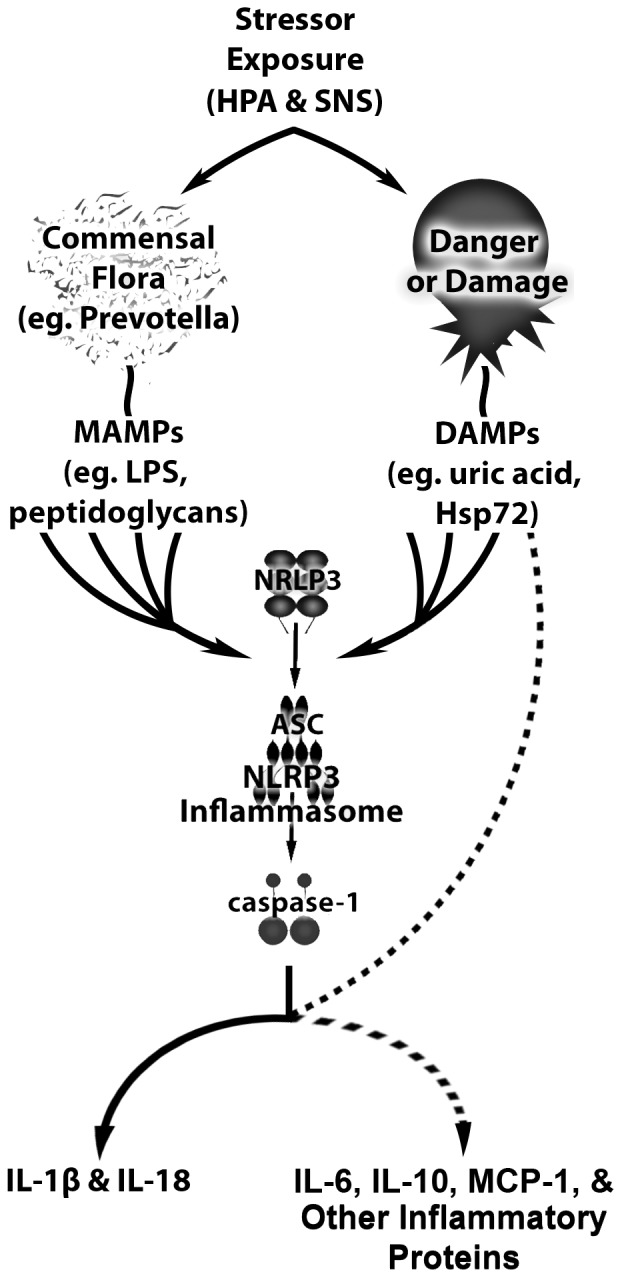
Exposure to a stressor activates the hypothalamic-pituitary-adrenal axis and sympathetic nervous system resulting in changes to the commensal flora (including a decrease in prevotella) and the release of microbe associated molecular patterns (MAMPs) as well as the the release of danger associated molecular patterns (DAMPs) either actively or via cell death. DAMP and MAMP signals then converge upon the inflammasome to yield IL-1β and IL-18 production. DAMPs may also act to drive responses from inflammasome independent inflammatory proteins including IL-6, IL-10, and MCP-1.

The present study is the first to demonstrate that release of stress-inducible inflammasome dependent inflammatory proteins depend on commensal bacteria. It is also the first to establish that commensal bacteria mediate acute stress-induced immune activity, and to directly demonstrate a role for MAMPs in this process. Each of these findings provides a novel mechanistic description of how exposure to stressors elevates blood concentrations of cytokines and chemokines. Furthermore, the aggregate of these findings alludes to a novel, inflammasomal pathway for stress-induced cytokine and chemokine production as summarized in **Figure 8**. Further examination of the interplay between the commensal bacteria and the inflammasome is important and may result in the development of therapeutic candidates that can suppress the cytokine storm evoked by severe stressors or trauma [70], [87], [88].
