# Correction: Commensal Bacteria and MAMPs Are Necessary for Stress-Induced Increases in IL-1β and IL-18 but Not IL-6, IL-10 or MCP-1

**DOI:** 10.1371/annotation/c7a5bcbd-3464-4e9e-bf74-e1aafe3f685b

**Published:** 2013-08-21

**Authors:** Thomas Maslanik, Kate Tannura, Lucas Mahaffey, Alice Brianne Loughridge, Lida Benninson, Luke Ursell, Benjamin N. Greenwood, Rob Knight, Monika Fleshner

The name of the fifth author is incorrect. The correct name is Lida Beninson. 

The correct citation is: Maslanik T, Tannura K, Mahaffey L, Loughridge AB, Beninson L, et al. (2012) Commensal Bacteria and MAMPs Are Necessary for Stress-Induced Increases in IL-1β and IL-18 but Not IL-6, IL-10 or MCP-1. PLoS ONE 7(12): e50636. doi:10.1371/journal.pone.0050636. 

